# Falls Induced by Optogenetic Inhibition of Basal Forebrain Cholinergic Projections after Dorsomedial Striatal Dopamine Depletion in a Dual Disruption Model of Falling Vulnerability in Parkinson Disease

**DOI:** 10.64898/2026.06.04.729642

**Published:** 2026-06-04

**Authors:** Aaron Kucinski

**Affiliations:** University of Michigan, Dept. of Psychology, 530 Church Street, Ann Arbor, MI 48103

## Abstract

Falls are a common and debilitating feature of Parkinson’s Disease (PD) patients. Prefrontal acetylcholine (ACh) deficits, as well as nigrostriatal dopamine deficits, are implicated in vulnerability to falls. PD patients with loss of cortical ACh and associated cognitive dysfunction experience a higher rate of falls than PD patients without cortical ACh loss. In addition, chemogenetic inhibition of basal forebrain (BF) projections in rats increases the vulnerability to falls on a balance beam task. Here, the impact of transient optogenetic inhibitions of BF-cortical cholinergic projections was assessed in rats with dorsomedial striatal dopamine lesions during traversal of straight or zig-zag balance beams in the Michigan Complex Movement Control Task (MCMCT). Adding transient optogenetic inhibition of BF-cortical cholinergic projections in rats with striatal dopamine lesions increased falls above the level produced by striatal dopamine lesions or BF ACh inhibition alone, especially on the challenging zig-zag task. These results support the critical role of BF-cortical cholinergic circuits in alleviating vulnerability to falls in PD patients with striatal dopamine loss, suggesting that it is combined loss of BF cholinergic projections and striatal dopamine that leads to greatest vulnerability to falls and related complex movement impairments.

## Introduction

Parkinson’s Disease (PD) is a multi-system neurodegenerative disorder that involves changes in multiple neurotransmitter systems. Loss of dopamine (DA) in the dorsal striatum is the principal neurotransmitter deficit responsible for the characteristic movement impairments. Changes in cholinergic systems also play key roles in disease pathophysiology (Bohnen et al., 2022). PD patients with regional cholinergic deficits often develop additional features including cognitive deficiencies (Bohnen et al., 2006; Schliebs & Arendt, 2011), as well as levodopa-resistant movement deficits such as impaired gait, poor balance and posture, and a higher propensity for falls than PD patients without cholinergic losses (Balash et al., 2005; Rochester et al., 2012). Falls are dangerous and a leading cause of death in PD patients and in the elderly in general, particularly in those with cognitive decline (Balash et al., 2005; Grimbergen et al., 2004; Holtzer et al., 2007). PD patients that fall tend to do so recurrently, especially patients with brain cholinergic deficits (Bohnen et al., 2009) and those with cognitive dysfunction such as poor attention (Allcock et al., 2009; Yarnall et al., 2011).

The basal forebrain (BF)-cortical cholinergic complex regulates cognitive processes (Gombkoto et al., 2021; Liu et al., 2015; Sarter & Paolone, 2011; Sarter & Lustig, 2020; Záborszky et al., 2018) and mediates the detection and integration of cues necessary to guide complex movement (Albin et al., 2022; Gritton et al., 2016). Degeneration of BF-cortical cholinergic pathways contributes to declining cognition in humans (Ballinger et al., 2016; Düzel et al., 2010) and rodents (Sarter, 1998). Loss of BF cholinergic neurons and reduced ACh release in the cortex has also been described in PD patients with impaired cognition (d’Angremont et al., 2025; Grothe et al., 2021; Schulz et al., 2018; Wolf et al., 2014), including poor attention (Woollacott & Shumway-Cook, 2002), and in patients with gait impairments such as high fall propensity (Dalrymple et al., 2021; LaPointe et al., 2010), particularly while traversing complex surfaces or dual-tasking (Kim et al., 2019).

We previously developed a rat model of high fall propensity and deficient control of complex movement using dual-system lesions that targeted both the DA and ACh neurotransmitter systems affected in PD (Kucinski et al., 2013; Sarter et al., 2014), as well as reversible chemogenetic deactivation of ACh systems using Designer Receptors Exclusively Activated by Designer Drugs (DREADDS) (Kucinski et al., 2019). For example, rats with partial losses of dorsal striatal dopamine terminals following striatal 6-OHDA infusions, combined with loss of cortical ACh after BF lesions, exhibited heightened propensity for falls on a challenging balance-beam traversal task, the Michigan Complex Movement Control Task (MCMCT), as well as reduced attentional performance on a sustained attention task (SAT). Decreasing cholinergic output from BF to the cortex in those studies was achieved either using a cholinergic-specific neurotoxin infused into the BF, or by using infusions of nonspecific DREADDs into BF to transiently turn off BF neuronal ACh output (Kucinski et al., 2013; Kucinski et al., 2019; Kucinski et al., 2022).

I sought to replicate and extend previous findings of elevated fall propensity from the combined ACh/DA losses, and to compare with less severe impairment induced by selective losses of either ACh or DA separately in isolation. Dual DA/ACh impairments were achieved in ChAT-Cre rats by combining striatal dopamine loss via 6-OHDA lesions with selective and reversible inhibition of BF cholinergic output using wireless LED optogenetics to transiently silence BF cholinergic projections on a sub-second time scale. Vulnerability to falls was assessed using the MCMCT as rats traversed either a rotating straight balance beam, or an even more challenging rotating beam shaped in a zig-zag pattern.

## Methods

### Subjects and Housing

ChAT-Cre heterozygous male and female Long–Evans rats between 2 and 3 months of age were obtained from Stanford University (Department of Bioengineering) and bred with wild-type (WT) Long–Evans female rats obtained from Envigo, and genotyped by Transnetyx using tail snips. Animals were individually housed in opaque single standard cages (27.70 cm × 20.30 cm) in a temperature- and humidity-controlled environment (23 °C, 45%) and maintained under a 12:12 h light/dark schedule (lights on at 7:00 AM). Food (Envigo Teklad rodent diet) and water were available *ad libitum*. All procedures were conducted in adherence with protocols approved by the University Committee on Use and Care of Animals at the University of Michigan and in laboratories accredited by the Association for Assessment and Accreditation of Laboratory Animal Care.

### Sign and Goal Trackers

Previous research found that disruption of ACh projections, induced by nonspecific chemogenetic inhibition of BF neurons, was more likely to increase falls by goal trackers (GT) than sign trackers (ST) (Kucinski et al., 2019). GT and ST rats were further compared here while testing the effects of dual ACh and DA disruptions produced by optogenetic ACh inhibition in BF combined with 6-OHDA loss of striatal dopamine. Sign-tracking is assessed in Pavlovian reward conditioning tests as a tendency to approach and engage with reward-associated cues (CS+) and has been suggested to be associated with relatively greater vulnerability for developing and maintaining addiction-like behaviors due to increased responsiveness to salient stimuli (Pitchers et al., 2017a; Pitchers et al., 2017b; Robinson et al., 2014; Yager & Robinson, 2013). Goal-tracking, on the other hand, is classified as approach directly to the location of rewards themselves, and is associated with sustained released of cortical acetylcholine during attention task performance (Paolone et al., 2013), elevated presynaptic choline uptake in BF cholinergic neurons relative to STs (Koshy Cherian et al., 2017), and a more top-down, cognitive approach that results in better performance on a sustained attention task (Kucinski et al., 2022).

#### GT/ST Screening.

Following genotyping, ChAT-Cre positive rats underwent Pavlovian Conditioned Approach (PCA) screening for individual propensity toward goal-tracking versus sign-tracking (Meyer et al., 2012). PCA screening yielded 27 GTs (15 females) and 30 STs (20 females) (N=57 total), which were used to assess the effects of optogenetic deactivation of cholinergic neurons of the BF on MCMCT performance measures. Rats were aged 3-4 months during PCA screening and 4-6 months during MCMCT testing. Following completion of the MCMCT test battery, rats were perfused for histological analyses.

### PCA Screening for STs vs GTs

#### Apparatus and procedures.

The screening of STs and GTs using a PCA test followed established and previously described methods (e.g., Paolone et al., 2013; Pitchers et al., 2017a; Yager et al., 2015). Briefly, rats were handled daily for 3 days and given ~15 banana-flavored grain-based pellets (45 mg; BioServ) in their home cages for 2 d prior to the start of PCA testing. Rats were tested in conditioning chambers (20.5 × 24.1 cm floor area, 20.2 cm high; MED Associates Inc.). Throughout the duration of the experiments, males and females were tested in separate chambers. A food magazine in the center of one of the walls of the chamber with an automatic feeder delivered banana pellets. Infrared photobeam breaks detected magazine entries. On either side of the magazine was a retractable lever with an LED backlight illuminated only when the lever extended into the chamber. Deflections of the lever were used to quantify lever contacts. The beginning of a test session was signaled by the illumination of a red house light located near the ceiling of the side of the chamber opposite to the magazine/lever. On the first day of testing (“pretraining”), rats were placed into the conditioning chambers and the house light was illuminated after a 5 min habituation period. Twenty-five pellets were then delivered on a VI-30 (0–60 s) schedule. On average, this pretraining session lasted 12.5 min and the lever was retracted throughout the session. During this session and all subsequent PCA sessions, rats consumed all pellets. The house light was turned on in the next five PCA sessions and rats were presented with 25 lever/pellet pairings delivered on a VI-90 (30–150 s) schedule. The CS for each trial was the extension of the illuminated lever into the chamber for 8 s. After lever retraction, a pellet was immediately delivered into the magazine. On average, the PCA test sessions lasted 37.5 min.

#### PCA measures and classification criteria.

Lever presses and magazine entries during the CS periods were used to quantify three measures of approach that determined the PCA index score. (1) Response bias was defined as the difference between lever presses and magazine entries, expressed as a proportion of the total responses [(lever presses–magazine entries)/(lever presses+magazine entries)]. (2) Latency score was calculated as the difference between the latency to approach the lever and the magazine after CS presentation; this difference was normalized by dividing by the maximum 8 s latency [(magazine latency–lever latency)/8]. (3) Probability difference was calculated as the difference between the probabilities of pressing the lever during the CS (i.e., the number of trials with a lever press out of 25 trials) minus the probability of entering the magazine. The PCA index score was the average of the response bias score, latency score, and probability difference. The values of this score ranged from 1.0 to −1.0, with a score of 1.0 indicating approaches and contacts of the lever on every trial, and a score of −1.0 indicating approaches and contacts of the magazine entry on every trial. Rats with an averaged PCA index score from PCA sessions 4 and 5 ranging from −1.0 to −0.4 were defined as GTs (i.e., rats more likely to direct behavior toward the food magazine than the lever), and rats with a PCA index score between +0.4 and +1.0 were designated as STs (i.e., rats more likely to direct behavior toward the lever-CS than the food magazine). Approach responses (response probability, number of contacts, and latency) were analyzed with repeated-measures ANOVAs with phenotype (STs, GTs) as the between-groups measure and training day as the within-subject factor.

#### Surgery.

Rats then underwent infusion surgeries, receiving bilateral microinjections of the AAV inhibitory virus (BF) or an inactive control plasmid, as well as neurotoxin 6-hydroxydoamine (6-OHDA) or sham infusions (striatum), followed by three weeks recovery, followed by a second surgery for implantations of optogenetic LED cannulae into the BF.

Rats were placed in vaporization chambers and anesthetized with 4–5% isoflurane delivered at 0.6 L/min O2 using a SurgiVet Isotec 4 Anesthesia Vaporizer until the animals were no longer responsive to a tail pinch and exhibited no hind limb withdrawal reflex. Heads were shaved using electric clippers and cleaned with a betadine scrub and alcohol pad. The animals were then mounted to a stereotaxic instrument (David Kopf Instruments) and isoflurane anesthesia was maintained at 1–3% for the remainder of the surgery. An ~2.5 cm incision was made down the midline of the scalp to expose the skull. The animals’ body temperature was maintained at 37°C using Deltaphase Isothermal Pads (Braintree Scientific). Ophthalmic ointment was used for eye lubrication. To prevent hypovolemia and hemodynamic instability, 1 mL/100 g 0.9% NaCl was administered subcutaneously. Animals also received an injection of an analgesic (carprofen; 5.0 mg/kg, s.c; Henry Schein Medical) before surgery and once or twice daily as necessary for 48 h postoperatively for pain relief.

The optogenetic viral vector containing plasmid pAAV-nEF-Con/Foff 2.0-iC++-EYFP was obtained from AddGene (Watertown, MA; AddGene plasmid #137156-AAV8). The adeno-associated virus (AAV) coding for a NEF-driven, Cre-dependent expression of chlorideconducting channelrhodopsin iC++-EYFP was stereotaxically infused into the BF of N=41 rats (N=25 Group 1, inhibitory AAV + 6-OHDA, and N=16 Group 2, inhibitory AAV + sham striatum), targeting primarily the fronto-medial cortical projection systems arising from the HDB. A control (inactive) vector containing plasmid pAAV-Ef1a-Con/Foff 2.0-EYFP (AddGene plasmid #137162) was infused into the BF of N=16 rats (Group 3, control AAV + 6-OHDA).

One μl of pAAV-nEF-Con/Foff 2.0-iC++-EYFP or pAAV-Ef1a-Con/Foff 2.0-EYFP (control) vector was infused (bolus) into the BF at two sites per hemisphere (AP −0.6; ML ± 2.4; DV: −7.6; AP −1.0; ML ± 2.9; DV: −8.0 mm). The injector was left in place for 8 min to minimize diffusion into the injector tract. In the same surgery, rats received bilateral bolus infusions (4.0 mg/ml;1 ml/hemisphere) of the neurotoxin 6-hydoxydopamine (Sigma-Aldrich; n = 41 rats; Group 1 and Group 3) or vehicle-only (1 mg/ml ascorbic acid in 0.9% NaCl; n = 16) into two sites of the dorsomedial striatum (AP +1.2; ML ± 2.2; DV: −4.5; AP +0.2; ML ± 2.5; DV: −5.0 mm). Rats were injected with desipramine hydrochloride (10 mg/kg, i.p.; Sigma-Aldrich) 30 min before 6-OHDA infusions for protection of noradrenergic neurons (Breese & Traylor, 1971). Rats were given 3 weeks to recover from the viral plasmid and 6-OHDA infusions.

In a second surgery, rats were anesthetized and prepared according to the parameters detailed above, and the LED cannulae with bilateral plastic fibers were implanted. Holes were drilled bilaterally on the skull approximately between the two viral infusion sites (AP −0.6 and AP −1.0) and the custom-sized cannulae were slowly lowered using a stereotaxic armpiece until the underside of the cannula units contacted the skull. Acrylic resin powder (Land Denture Mfg. Co. Inc.) and self-curing liquid resin (Ortho-Jet Liquid) were used to secure the cannulae in place as well as to adhere C-shaped 3D-printed plastic pieces for the attachment of clips to secure the receiver to the prongs of cannulae during traversals. Behavioral testing commenced after one week of recovery.

### LED Wireless Optogenetic System

In earlier pilot experiments on traditional optogenetic techniques using fiber optic cables to deliver laser illumination, it was found that connecting fiber optic cables to rats’ headstages during balance beam tasks could restrict their movement and disrupt performance, as well as being prone to lose connection if a rat accelerated quickly while traversing a rod, or if it fell from the MCMCT rods. Thus, a wireless LED optogenetic system was used for these experiments (Teleopto Wireless Optogenetic Stimulator System; Amuza Inc.; San Diego, CA). This light-delivering system comprised a wireless receiver (attached to the animals’ headstages), implanted custom-sized fiber optic LED cannulae (TelLCD-B-7.0-250-5.5, 470 nM, 5.59 mW; Amuza Inc.), a remote controller and an infrared emitter to initiate pulse generator LED stimulation (5 V) from a distance of up to 2 meters (STO mk II; Azuma Inc.).

Rats were implanted with bilateral LED cannulae (470 nm) with plastic fibers, custom-sized to reach the basal forebrain region just above the optogenetic virus infusion sites (7.0 mm length; 5.5 mm between fibers; 250 μm fiber diameter). On test days, the wireless receiver (TeleR-1-P; 13 x 18 x 7 mm; weighing 1.4 g) was attached to connection prongs of the cannulae on the rats’ headstages and secured in place with custom-made clips. The LED fibers were activated manually by a remote controller relay and pulse stimulator. Pulses were either 1s in duration or active for the duration of traversals in continuous stimulation runs.

#### MCMCT Test Battery.

57 ChAT-Cre+ rats, 27 GTs (15 females) and 30 STs (20 females), were assigned pseudorandomly to three groups prior to surgeries: (1) inhibitory AAV + 6-OHDA (Group 1 or ‘*Dual Loss ACh/DA’*; N = 25; 7 GT females, 5 GT males; 8 ST females, 5 ST males); (2) inhibitory AAV + sham striatum or ‘*ACh Loss Only’* (Group 2; N = 16; 4 GT females, 4 GT males; 5 ST females, 3 ST males); and (3) control AAV + 6-OHDA or ‘*DA Loss Only’* (Group 3; N = 16; 4 GT females, 3 GT males; 5 ST females, 4 ST males). Following surgeries and recoveries, rats underwent a 17-day sequence of MCMCT sessions (see Table 1), beginning with a 3-day training block of runs on the plank, straight stationary (non-rotating) rod and straight rotating rod (Days 1-3). Next, rats traversed the straight rotating rod for a block of three consecutive sessions, receiving 1s inhibitory LED pulses (Days 4-6). Rats were then habituated to the zig-zag rod in two practice sessions, stationary on Day 7 and rotating on Day 8, followed by a block of three test days on the rotating zig-zag rod with 1s inhibitory LED pulses (Days 9-11). After a one-week break with no MCMCT sessions, rats underwent blocks of testing on the straight rotating rod (Days 12-14) and the zig-zag rotating rod (Days 15-17) as LED inhibition was delivered continuously throughout the duration of LED runs.

All rats that completed testing on the straight rotating rod with 1s LED pulses (Days 5-7) also completed testing on the straight rotating rod with continuous pulses (Days 9-11), except for 5 rats from early experiments that were only tested with 1s pulses on both the straight and zig-zag rods (all from group 1). Some rats were removed from zig-zag rod analyses due to the inability or refusal to traverse - one group 1 rat (male, ST) did not complete zig-zag runs with 1s pulses and 3 rats (2 males - group 1 GT and group 2 GT and 1 female - group 2 GT) did not complete zig-zag runs with continuous pulses. In addition, rats with abnormally high baseline (no LED) fall rates on the zig-zag rotating rod (>0.75 falls per run) were removed from the analyses so that effects of inhibitory pulses could be more clearly assessed (1s pulse runs: 9 males removed (group 1: 2 GT and 1 ST; group 2: 3 GT and 2 ST; group 3: 1 GT) and 1 female removed (group 3: 1 ST); continuous pulse runs: 10 males removed (group 1: 1 GT, 1 ST; group 2: 2 GT and 2 ST; group 3: 1 GT and 3 ST) and 1 female removed (group 3: 1 ST)). The final N values for each condition are included in the results section.

#### Optogenetic Testing Parameters.

LED-induced optogenetic inhibition of BF cholinergic neurons was first assessed by delivering two 1s pulses per traversal on the straight rotating rod (Days 4-7). In this block of testing, rats performed 8 endbox-to-endbox traversals of the 3-meter rod per day, 4 with LED inhibitory pulses delivered twice per traversal, and 4 traversals in which no pulses were delivered. Pulses were activated manually by the experimenter when the rat crossed the 0.5-meter and the 1.5-meter demarcations enroute to the endboxes. Each pulse was 1s in duration. Thus, per test day, rats received 8 inhibitory pulses over 4 traversals (and for each 3 test-day block, 24 inhibitory pulses over 12 traversals). In the corresponding no LED traversals, no pulses were delivered. Pulses were given in two consecutive traversals - in traversals 1 and 2 and 5 and 6 (with no pulses in traversals 3 and 4 and 7 and 8) or traversals 3 and 4 and 7 and 8 (with no pulses in traversals 1 and 2 and 5 and 6). The order was randomized across all animals and conditions.

### Balance Beam task: the Michigan Complex Movement Control Task (MCMCT)

The MCMCT beam traversal apparatus (Kucinski et al., 2013; Kucinski & Sarter, 2021) was used to assess traversal performance measures in rats performing attention-demanding crossings of a ‘straight’ narrow rod or an angled rod surface (‘zig-zag’). The rods were 3.0 m in length (square; side lengths 1.59 cm), made from aluminum tubing covered with gray gaffer’s tape for traction. There was also a plank surface (13.3 cm width) placed directly on top of the straight rod and fitted firmly in place inside edges of the support towers used to habituate rats to traverse from endbox to endbox. The straight and zig-zag rods could be rotated using a 12 VDC electric motor controlled remotely by a pulse width modulator which was able to adjust the speed of rotation (up to 10 RPM) and switch the direction of rotation. A safety net was suspended 20 cm under the beam to catch the rats during falls. Two identical endbox stations were situated on top the support towers at opposite ends of the beam. These stations consisted of a 30 x 25 cm platform and were surrounded by a retractable wall structure (23 cm height in raised position) to allow conversion from an open platform to a boxed structure. On the wall facing the beam of each box structure were 9-cm wide openings that allowed the rats access to and from the beam. The walls were raised and lowered mechanically with a 12 VDC electric motor actuated remotely by a toggle switch. Lowering the walls and thereby turning the box into an open field platform was sufficient for rats to initiate beam traversal to enter the opposite endbox (with walls up). Between traversals, the walls of the endbox remained in the up position for 45 s. Videos of traversals were recorded with 8 bullet Ubiquiti Networks UVC-G4-PRO UniFi cameras (50P frame rate/8 MP resolution/3840x2160/3x zoom) mounted to the net frames parallel to both sides of the beam, 4 per side, to provide visualization of rats from both sides during traversals. Also attached to one side of the net frame, adjacent to the cameras, were LED indicator lights (red) activated by the pulse modulator to signal on video the precise timing of the pulse deliveries. The camera feeds were transmitted to a PC using a video capture system (Inmotion Studios, Ann Arbor, MI; UNVR with 8TB HDD Network Video Recorder Pro, 16 port giga ethernet POE switch, WD surveillance hard drive Raid1, cloud controller for UnFi camera system) and visualized in two continuous feeds (left and right sides) using customized video software (Arbor Computers, Ann Arbor, MI).

#### Straight rotating rod.

Falls and traversal time were the measures of traversal performance on the straight rotating rod. These measures were analyzed in the same manner as previously described (Kucinski et al., 2019; Kucinski & Sarter, 2021). During falls, rats either fell into the net and were placed back onto the rod or, upon an imminent fall, the experimenter assisted the rat in regaining balance on the rod, and no further falls were counted until the rat regained a balanced posture and resumed forward movement. In addition to complete falls from the straight rod, instances in which the rats ceased forward movement and assumed a slouched, nearly immobile position on the rod were counted as falls. In either case, traversal time was corrected for fall-related disruptions of forward movement. In most instances, rats were able to regain forward movement within 2 s of being placed back onto the rod. Since the 1s LED pulses were not delivered until rats reached 0.5 m along the 3.0 m rod, falls that occurred prior to that point were not counted in both LED and no LED runs, and counting of traversal time commenced at the 0.5 m mark along the beam. In runs in which continuous LED pulses were given, performance measures were scored over the duration of the traversals. Also, since the 1s pulses were delivered twice per run (when rats approached 0.5 m and 1.5 m along the rod), a maximum of one fall per pulse or section of the rod (from 0.5 m to 1.5 m or 1.5 m to 3.0 m) was counted. Thus, a maximum of 2 falls per full traversal could be scored. To maintain consistency between testing conditions, a maximum of 1 fall per section of the rod (and 2 for each run) was also scored for continuous pulse runs. We previously reported high inter-rater reliability for extracting the behavioral measures from videorecorded MCMCT runs (Avila et al., 2020).

#### Zig-zag rotating rod.

A particularly challenging zig-zag rod variation was designed to determine the propensity for falls as rats performed traversals that taxed additional attentional control and flexibility of movement (Kucinski et al., 2018; Kucinski et al., 2019; Kucinski & Sarter, 2021). This rod featured two zig-zag sections, each 0.6 m in length, placed 0.6 m from either end of the rod and separated by a 0.6-m long straight rod in the middle. Each zig-zag section included three angled sections of varying lengths (two 10.7 cm and one 21.5 cm in length) that bended at 45° angles from the horizontal plane of the rod and connected to two straight sections (each 15 cm in length) that rested 3 cm above or below the plane the vertical plane of the rod. The diameter of the rod tube remained 2.54 cm^2^ across the entire rod. The 1s LED pulses were delivered as the rats initiated forward movement onto the bent sections (2 per run). Falls and traversal time were the performance measures assessed on the zig-zag rotating rod runs. As done previously (Kucinski et al., 2019), one fall maximum was scored per zig-zag section (2 per run).

#### Scoring behavioral performance.

Fall propensity from the straight rod was assessed by determining if a fall occurred during the period in which the rat first received an inhibitory pulse until either the rat received a second pulse (from the 0.5-meter to 1.5-meter demarcation) or until the end of each traversal (from the 1.5-meter demarcation to the endbox). The same criteria were used to count falls when no pulses were delivered. Thus, per traversal, there was a maximum of 2 falls scored, per test day a maximum of 8 falls, and per 3-day test block a maximum of 24 falls in LED runs. Fall totals over the 3-day block was scored out of a possible of 24 falls with inhibitory pulses and 24 possible falls with no pulses. In the next block of traversals, rats crossed the zig-zag rotating rod. Pulses were delivered when the front paws of the rats stepped onto the zig-zag sections as the rat moved toward the endbox, at approximately 1 and 2 meters along the rod. Fall totals were similarly scored with a maximum of 1 fall per pulse (or per zig-zag section), two per traversal, 8 per test day, and 24 for the block (with a maximum of 24 possible falls in no LED runs). Falls were also counted with continuous inhibitory pulses in two 3-day blocks, one on the straight rotating rod and one on the zig-zag rotation rod. For these runs, the LED pulses were activated for the entire duration of traversal - from when the rats first initiated traversal onto the rod until stepping into the endbox on the far end of the rod. Falls were counted in the same manner as the 1s pulse runs, with a maximum of two per traversal, one in first part of each run (either from the 0.5 to 1.5-meter demarcations on the straight rod or across the first zig-zag portion) and one in the second part (either from the 1.5 demarcation to the endbox on the straight rod or the across the second zig-zag portion). A maximum of 24 falls in LED and no LED runs could be scored for continuous inhibition runs as was with runs with 1s pulses.

### Histological visualization and quantification of AAV transfection space and 6-OHDA lesions

Following the end of all MCMCT tests, rats were deeply anesthetized with a lethal dose of sodium pentobarbital (270 mg/kg, i.p.) and transcardially perfused with phosphate buffer solution (PBS), followed by 4% paraformaldehyde in 0.15 M sodium-phosphate solution, pH 7.4. Brains were extracted and post-fixed in 4% paraformaldehyde for 24 hrs, rinsed with PBS and then placed in 30% sucrose solution until they sank. Brains were sectioned into 35-μm thick slices using a freezing microtome (CM 2000R; Leica) and stored in cryoprotectant until further histological processing.

To determine the expression space of the AAV in the BF, sections were double-stained to both amplify the signal of the eYGFP fluorescent tag as well to visualize cholinergic neurons, using antibodies against the vesicular acetylcholine transporter (VAChT; Arvidsson et al., 1997). Sections were put in 0.1 M PBS (pH 7.3–7.4) overnight the day before staining. The next day sections were rinsed 3 times for 5 mins in 0.1 M PBS, then blocked in 0.1 Triton X100 diluted in PBS for 15 mins. Sections were then rinsed in PBS 3 times for 5 mins and incubated with 1% Normal Donkey Serum (NDS) and 1% Triton X100 made in PBS for 60 min at RT. The sections were then incubated overnight in the primary antibodies (chicken anti-GFP; 1:2000; Abcam, ab167453; and goat anti-VAChT; 1:500; Synaptic Systems, Cat. No. 139 103) in a cold room. The following day, sections were first rinsed 3 times for 5 each with PBS, and then incubated in secondary antibodies (Alexa 488-conjugated donkey anti chicken; 1:300; Jackson Laboratories, Cat. No. 703–545-155) and Alexa 594-conjugated donkey anti rabbit; 1:500; Jackson Laboratories, Cat. No. 711–585-152) in PBS, 0.5% normal donkey serum and 0.5% TritonX100 for 90 mins at RT. Sections were rinsed with PBS 3 times for 5 mins and then mounted and cover-slipped with Vectashield Antifade Mounting Medium (H-1000; Vector Laboratories). A Zeiss LM 700 confocal microscope was used to inspect and photomicrograph fluorescent neurons at 10X (for cell count estimates) and 20X (for verifying and documenting double-labeled cells) at two A-P levels (−0.72 and −0.96 mm). The microscope was equipped for sequential multi-track acquisition with 488 nm and 561 nm excitation lines and filter sets specific for Alexa 488 (Zeiss filter set 38 HE) and Alexa 594 (Zeiss filter set 54 HE), respectively. Single- and double-labeled neurons were counted in three subregions of the basal forebrain (nucleus basalis of Meynert, nbM; horizontal limb of the diagonal band, HDB; substantia innominata, SI). Using the ImageJ multipoint tool, superimposed counting frames were generated for each subsection (approximate sizes, actual subregion areas were based on irregular shaped outlines from the rat brain atlas: nbM: 0.4 mm x 0.8 mm; HDB:1.4 mm x 1 mm; SI: 0.5 mm x 0.9 mm) and counts were restricted to these frames. Counts were generated based on 2 sections per rat (−0.80 and −1.00 mm from bregma), yielding 4 counts per rat and subregion. Correlations of counts with falls were most apparent in the more posterior (−1.00 mm) sections, thus these are reported in the results section. Tile scanning/stitching was utilized at the 10X (4x4 tiles, 2368.77 by 2368.77 μm). The split view on Zen Black software was used to view single channels of the multi-track shots. Cells with green (Alexa 488) and red (Alexa 594) fluorescence located primarily in the soma were used in counts for double-labeled neurons.

To visualize the size and extent of 6-OHDA lesions, TH immunostaining was performed using a primary antibody (rabbit anti-TH; ab112, abcam), Vectastain Elite ABC Kit (PK-6101, Vector Laboratories) and DAB Substrate Kit (SK-4100, Vector Laboratories). Sections were first rinsed 3 times for 3 mins with 0.1 M PBS (pH 7.4) and then incubated in 0.3% H2O2 for 30 min. Sections were rinsed 3 times in 0.5% Triton (in PBS) for 3 mins each and then incubated in a blocking buffer (Vectastain buffered NGS + 0.3% Triton in PBS) for 1 hr at RT. Sections were then rinsed 3 times for 3 mins in PBS and incubated for 24-48 hrs with primary antibody (1:1000; 0.3% Triton in PBS) in a cold room. Sections were then rinsed 3 times for 3 mins with 0.3% Triton in PBS, incubated for 30 mins in secondary antibody (biotinylated goat-anti rabbit; 1:500, Vectastain) and then washed in PBS for 3 mins in 3 rinses. Sections were then incubated in ABC Elite (1:1000) for 30 mins. After another 3 rinses in PBS for 5 mins each, sections were developed in DAB substrate solution (buffer, DAB, H2O2, nickel) until they appeared dark (2-5 mins), and then rinsed 3 times in PBS for 5 mins. Sections were mounted with gelatin on SuperFrost Plus slides and allowed to dry overnight. The next day, the slides were dehydrated in an increasing alcohol series (70%, 95%, and 100%) and defatted in xylenes before coverslipping. Sections were inspected, and images were taken with a Leica DM400B digital microscope.

Photographs of TH immuno-stained sections in Group 1 rats (inhibitory AAV + 6-OHDA) taken at 1.25X from sections at approximately 500 μm posterior and anterior from 0.2 AP to 1.2 AP - the sites of infusion. The extent and location of the bleaching of TH stains was rated. TH-IR losses greater than 2.00 mm (dorsal-ventral plane) x 0.5 mm (medial-lateral plane) x 0.5 mm (anterior-posterior plane) were assigned the highest score (5). Lower scores (<3) were assigned to smaller lesion sizes. We previously determined that TH losses in the mediodorsal part of the striatum, overlapping with the target field of prefrontal cortical projections are necessary and sufficient to produce falls in rats with BF cholinergic lesions (Kucinski et al., 2013; Kucinski et al., 2015). TH losses that were centered closest and that were mostly restricted to the mediodorsal caudate nucleus were assigned the highest score (5), whereas lesions spreading more laterally received lower scores. The size and placements scores for both hemispheres were averaged to a single DA lesion score (maximum of 5) for each rat.

### Statistical analyses

The effects of inhibitory 1s or continuous pulses on MCMCT performance measures were compared between the three experimental groups (group 1: inhibitory AAV + 6-OHDA or ‘*Dual Loss ACh/DA,’* group 2: inhibitory AAV + vehicle striatum or ‘*ACh Loss Only’*, group 3: control AAV + 6-OHDA or ‘*DA Loss Only’*) using mixed measures ANOVA. Between-subjects factors Sex and Phenotype (ST or GT) and within-subjects factor Day (within each 3-day block) were used when applicable. *Post hoc* pairwise comparisons were conducted using Fisher’s Least Significance Difference (LSD) test. Statistical analyses were performed using the SPSS for Windows (version 17.0: SPSS). Assumptions underlying the statistical model were assessed. In cases of violation of the sphericity assumption, Huyhn–Feldt-corrected *F* values, along with uncorrected degrees of freedom, are given. Alpha was set at 0.05 except for the analysis of falls. Exact *P* values are reported as recommended previously (Greenwald et al., 1996).

## Results

### Straight Rotating Rod, 1s inhibitory pulses.

Overall effects of optogenetic BF cholinergic inhibition on MCMCT performance measures on the straight rotating rod were first assessed in N=57 rats over a 3-day block 8 traversals per day (24 traversals total). In straight traversals, optogenetic 1s inhibitory pulses were delivered twice per optogenetic run (or no pulses in baseline no-inhibition runs). Optogenetic inhibition versus No inhibition was the within-subjects factor, and between-subject status factor was Disruption group status (either *Dual Loss ACh/DA*: active inhibitory optogenetic virus + 6-OHDA striatal lesions, N = 25; or *ACh Loss Only*: active inhibitory optogenetic virus + sham lesion striatum, N=16; or *DA Loss Onl*y: inactive eYFP control virus + 6-OHDA striatum lesions, N = 16). In this and subsequent analyses, GT/ST status and F/M sex were not significant factors or interactions, and so for simplification F/M rats and GT/ST rats were combined, and only the factors Optogenetic inhibition and Disruption group are reported. Since a maximum of 1 fall was scored per 1s pulse (or per section of the rod), a maximum of 8 falls per day or 24 for the 3-day block was scored, and total falls per block are reported out of a maximum of 24. Traversal times are reported per run (one endbox to endbox traversal).

Falls were significantly elevated overall on the straight rotating rod by optogenetic inhibitory 1s LED pulses (F(1,54) = 32.65, p < 0.001; no LED: 4.58±0.36 falls per rat over the 3-day block, LED: 6.47±0.48 falls). Although this did not interact significantly with group (F(2,54) = 1.53, p = 0.23), pairwise comparisons suggested that optogenetic 1-s pulses caused a significant increase in falls by the *Dual Loss ACh/DA group* (F(1,24)=23.05, P < 0.01; no-LED 4.68±0.39 falls, LED: 7.04±0.73), and by the *ACh Loss Only* group (F(1,15)=14.55, P < 0.01; no-LED: 4.31±0.58, LED: 6.31±0.72;), but only marginally by the *DA Loss Only* group (F(1,15)=3.35, P = 0.09; no-LED: 4.69±0.97, LED: 5.75±1.04). Falls overall significantly differed between test days, and progressively declined over the 3-day block (F(2,108)=21.45, P < 0.001): Falls on Day 1 (4.88±0.44 falls per day) were higher than on Day 2 (3.40±0.28; F(56)=4.05, P < 0.001) or Day 3 (2.78±0.26; F(56)=4.80, P < 0.001) and falls on Day 2 were also higher than Day 3 (F(56)=2.61, P = 0.01).

Traversal time was slower in LED runs (F(1,54) = 29.88, p < 0.001; no LED: 11.71±0.54 s per run; LED: 12.89±0.64), however this also did not significantly interact with group (F(2,54) = 0.14, p = 0.94). Pairwise comparisons indicated ACh inhibition slowed traversals by the *Dual ACh/DA Loss* group (no LED: 11.74±0.65 seconds per traversal, LED: 12.91±0.89; F(1,24)=10.85, P < 0.01) as well as by the *ACh Loss* Only group (no LED: 11.08±1.15 s, LED: 12.29±1.33; F(1,15)=8.91, P < 0.01), but did not significantly alter traversal time in the control *DA Loss Only* group (no LED: 12.30±1.23 s, LED: 13.44±1.28; F(1,15)=2.82, P = 0.11).

### Straight rotating rod, continuous inhibition.

Continuous inhibitory LED pulses also elevated falls overall on the straight rotating rod overall (N=52; group 1: N = 20; group 2: N = 16; group 3: N = 16) (F(1,49) = 23.09, p < 0.001; no LED: 3.90±0.36 falls per rat over the 3-day block, LED: 5.46±0.44 falls), and optogenetic stimulation interacted significantly with group (F(2,49) = 5.35, p = 0.008). Pairwise comparisons indicated that continuous ACh inhibition increased falls in the *Dual Loss ACh/DA* group (no-LED: 3.45±0.61 falls, LED:6.20±0.76 falls, F(1,19)=57.47, P < 0.001), but only marginally increased falls in the *ACh Loss Only* group (no LED: 4.38±0.57 falls, LED: 5.56±0.90 falls; F(1,15)=3.70, P = 0.07), and failed to have any effect in the *DA Loss Only* group (no-LED: 4.00±0.70 falls, LED: 4.44±0.52 falls; F(1,15)=0.73, P = 0.41). Falls did not differ significantly between test days within the 3-day block (Day 1: 3.19±0.33 falls; Day 2: 3.04±0.29; Day 3: 3.13±0.34; F(2,98)=0.60, P=0.94).

Traversal time was significantly slowed in LED inhibition runs (F(1,49) = 4.91, p = 0.03; no LED: 8.92±0.51 s per run, LED: 9.49±0.63 s). In particular, pairwise comparison indicated that ACh inhibition slowed traversals by the *Dual ACh/DA Loss* group (no LED: 8.71±0.82 s, LED: 9.64±1.09; F(1,19)=5.84, P = 0.03), but did not affect traversal time by the *ACh Loss Only* group (no LED: 8.28±0.81 s, LED: 8.27±0.79; F(1,15)=0.011, P = 0.99) nor the control *DA Loss Only* group (no LED: 9.84±1.05 s, LED: 10.53±1.29; F(1,15)=1.73, P = 0.21).

### Zig-zag rotating rod, 1s inhibitory pulses.

Falls and traversal time were next assessed on the more challenging zig-zag rotating rod (Total N=47 rats; *Dual ACh/DA Disruptio*n group: N = 22, *ACh Loss Only* group: N = 11, *DA Loss Only* group: N = 14). Presenting 1s ACh inhibitory LED pulses caused an overall increase in falls (F(1,44) = 26.48, p < 0.001; no LED: 5.74±0.30 falls per rat over the 3-day block, LED: 8.40±0.50 falls), an effect which differed across groups (LED x group interaction: F(2,44) = 5.26, p = 0.009). Pairwise comparisons indicated that 1-s optogenetic ACh inhibitions increased falls in the *Dual ACh/DA Disruptio*n group (F(1,23)=28.70, P < 0.01; no LED: 5.82±0.48 falls, LED: 9.95±0.80), but had only marginal effects in the *ACh Loss Only* group (F(1,15)=4.37, P = 0.054; no LED: 5.82±0.30, LED: 7.27±0.78), and no detectable effects in the *DA Loss Only* group (F(1,15)=0.84, P = 0.38; no LED: 5.57±0.65, LED: 6.86±0.50). Across groups, there were significantly more falls on the zig-zag rod in the *Dual ACh/DA Disruptio*n group than in either the *ACh Loss Only* group (p = 0.03; main effect of group: F(2,44) = 2.76, p = 0.08) or the *DA Loss Only* group (p=0.006), and the latter two groups did not differ from each other in falls (p = 0.75). Falls overall significantly differed between test days within the 3-day block, declining slightly over days (F(2,88)=7.09, P = 0.001). Falls on Day 1 (5.70±0.38 falls per day) were higher than on Day 3 (4.02±0.36; F(46)=2.43, P = 0.02) but not Day 2 (4.43±0.32; F(46)=1.43, P = 0.16), and falls on Day 2 and Day 3 did not differ (F(46)=0.87, P = 0.39).

Overall, rats traversal time was slower during LED inhibition runs (F(1,44) = 9.54, p = 0.003) and there was a main effect of group (F(2,44) = 5.79, p = 0.006). However, pairwise comparisons did not indicate significant slowing within the *Dual ACh/DA Loss* group by itself (no LED: 20.25±1.49 seconds per traversal, LED: 21.08±1.58; F(1,21)=2.42, P = 0.14), the *ACh Loss Only* group (no LED: 18.61±2.13 s, LED: 19.30±2.44; F(1,10)=0.87, P = 0.37) nor the *control DA Loss Only* group (no LED: 27.65±2.67 s, LED: 30.11±2.84; F(1,13)=4.05, P = 0.07) were slowed significantly by ACh inhibition.

### Zig-zag rotating rod, continuous inhibition.

With continuous LED inhibition on the zig-zag rotating rod (N=37; *Dual Loss ACh/DA*: N = 16, *ACh Loss Only*: N = 10, *DA Loss Only*: N = 11), falls were elevated overall compared to no-LED runs by the same rats (F(1,34) = 12.35, p = 0.001; no LED: 4.95±0.40 falls per rat over the 3-day block, LED: 6.82±0.63 falls), but this effect differed across groups (LED x group interaction: F(2,34) = 9.55, p = 0.001). In particular, pairwise comparisons indicated that continuous inhibition of ACh neurons caused increases in falls by the *Dual ACh/DA Loss* group (no LED: 5.06±0.62 falls, LED: 9.44±1.00; F(1,17)=25.73, P < 0.001), but only a marginal effect in the *ACh Loss Only* group (no LED: 3.70±0.80, LED: 5.70±0.63; F(1,13)=3.72, P = 0.08), and no detectable LED effect in the control *DA Loss Only* group that lacked an active BF optogenetic virus (no LED: 5.91±0.61, LED: 5.00±0.73; F(1,15)=0.51, P = 0.49). There were significant differences in falls across groups (no LED runs: F(2,36) = 2.19, p = 0.13; LED runs: F(2,36) = 7.78, p = 0.002). That is, *Dual ACh/DA Loss* rats with the active virus + 6-OHDA striatal lesions fell more than *ACh Loss Only* rats with the active virus + sham striatal lesions (p = 0.005) or *DA Loss Only* rats with the control virus + 6-OHDA striatal lesions (p=0.001). The *ACh Loss Only* and *DA Loss Only* groups did not differ in falls (p = 0.45). Falls overall significantly differed between test days within the 3-day block, declining slightly over days (F(2,68)=4.38, P = 0.02): Falls on Day 1 (4.49±0.41 falls per day) and Day 2 (4.32±0.37) did not differ (F(36)=0.45, P = 0.66), but falls on both Days 1 and 2 were significantly higher than on Day 3 (3.24±0.27; F>2.62, P < 0.02 for both).

Overall, traversal times were slower on LED inhibition runs (F(1,34) = 5.10, p = 0.03), however the group x LED interaction was not significant (F(2,34) = 0.34, p = 0.72; main effect of group F(2,34) = 0.86, p = 0.43). Pairwise comparisons indicated that ACh inhibition slowed traversals by the Dual ACh/DA Loss group (no LED: 17.97±2.06 s, LED: 19.56±2.23; F(1,15)=7.88, P = 0.01), but not by the ACh Loss Only group (no LED: 16.17±3.28 s, LED: 17.38±3.42; F(1,9)=1.09, P = 0.32) nor the control DA Loss Only group (no LED: 22.00±3.40 s, LED: 22.62±3.42; F(1,10)=0.83, P = 0.55).

### Lack of Sign Tracker vs Goal Tracker differences.

Potential GT/ST differences on falls and traversal times were assessed in a separate analysis, using LED inhibition (LED) versus No inhibition as the within-subjects factor, and GT/ST, sex, and Disruption group status (either *Dual Loss ACh/DA*, *ACh Loss Only,* or *DA Loss Onl*y) as between-subject factors. GT and ST rats did not differ in falls on any rod condition, nor were there any significant 2,3 or 4-way interactions involving GT/ST as a factor with LED or sex (F<2.33, p>0.11 for all). In the *Dual Loss ACh/DA* group, falls did not differ between GTs and STs on the straight rotating rod with 1s LED inhibition (overall GT/ST effect: F(1,23)=0.02, p = 0.91; LED x GT/ST interaction: F(1,23)=0.04, p = 0.84) nor with continuous LED inhibition (overall GT/ST effect: F(1,18)=0.75, p = 0.13; LED x GT/ST interaction: F(1,18)=2.49, p = 0.13). Similarly, on the zig-zag rotating rod, falls did not differ between GTs and STs with either 1s LED inhibitions (overall GT/ST effect: F(1,20)=0.01, p = 0.98; LED x GT/ST interaction: F(1,20)=0.47, p = 0.50) or with continuous LED inhibition (overall GT/ST effect: F(1,14)=0.16, p = 0.70; LED x GTST interaction effect: F(1,14)=0.16, p = 0.69).

In traversal times also, GTs and STs did not differ on any rod condition, nor where there any significant 2,3 or 4-way interactions involving GT/ST status with LED inhibition or with sex (F<2.46, p>0.10 for all).

### Sex Difference Effects.

On the straight rod, females and males did not differ in falls, nor where there any significant interactions between sex and 1s/continuous duration condition or LED inhibition condition (1s LED inhibition: F(1,51) = 0.36, p = 0.55 (sex x LED, sex x group, and 3way interaction, F < 0.58, p > 0.45 for all; overall mean (LED + no LED): females: 11.48±0.91 falls per 3-day block, males: 10.54±1.34; continuous LED inhibition: F(1,46) = 0.01, p = 0.95 (sex x LED, sex x group, or 3-way interaction; F < 1.06, p > 0.32 for all; overall mean (LED + no LED): females: 9.46±1.01 falls per 3-day block, males: 9.27±1.08).

However, on the more challenging zig-zag rod, larger males generally fell more often than females regardless of LED or no-LED condition, and there was no significant interaction (1s LED inhibition: F(1,41) = 6.23, p = 0.02 (sex x LED, sex x group, and 3-way interaction, F < 1.69, p > 0.20 for all; overall mean (LED + no LED): females: 12.94±0.81 falls per 3-day block, males: 16.73±1.00); continuous: F(1,31) = 15.87, p < 0.001 (no sex x LED, sex x group, or 3 way; F < 1.37, p > 0.26 for all; overall mean (LED + no LED): females: 10.04±0.72 falls per 3-day block, males: 16.82±1.41). The larger body size of male rats (417.53±32.42 g in 22 male rats vs 320.98±19.72 g in 35 female rats) was likely the primary cause of greater overall propensity of males to fall, as male rats have more trouble regaining upright posture following slips on the rotating rod.

In traversal time, larger males were generally also slower than females across all 4 traversal conditions, with no significant interactions (main effect of sex, F > 16.92, P < 0.001 for all; overall means: straight rod: 1s LED inhibition: females: 10.22±0.57 s, males: 15.16±0.86; continuous LED inhibition: females: 7.39±0.58, males: 11.33±0.82; zig-zag rod: 1s LED inhibition: females: 18.79±1.18 s, males: 31.09±1.98; continuous LED inhibition: females: 18.74±1.73, males: 27.64±2.27; Interactions of Sex with LED condition or 1s/continuous condition: F<1.54, p>0.22 for all).

### Histological Analysis.

#### Basal Forebrain.

**Figure 3.**
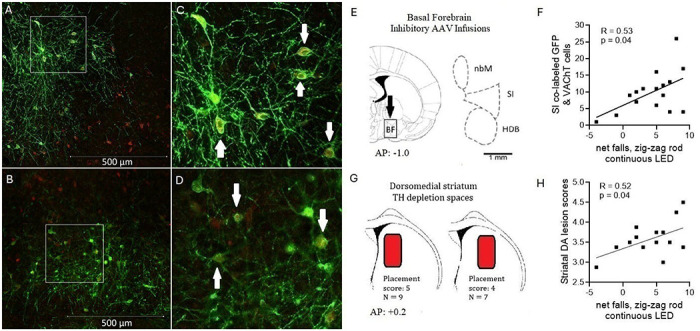
Visualization of the chloride-conducting channelrhodopsin iC++-EYFP in the BF (SI) of cholinergic and noncholinergic neurons (Alexa 594, appearing green, indicates the expression of the optogenetic viral construct (GFP amplified tag); Alexa 488, appearing red, indicates the presence of the VAChT in cholinergic cells). Low-magnification photomicrographs (500-μm scale inserted) of a coronal section showing the SI of a high falling rat with the inhibitory AAV + 6-OHDA lesion (15 falls in 24 trials on the zig-zag rod with continuous LED inhibition) (A) and a low falling rat with the inhibitory AAV + 6-OHDA lesion (B) (6 falls in 24 trials). These photomicrographs exemplify the dense presence of magnocellular cholinergic neurons (red) as well as numerous neurons expressing GFP or co-labelled cells. The areas marked by a whitish overlay in (A) and (B) are magnified in (C) and (D), respectively, and show several double-labeled neurons (arrows). More robust expression of the inhibitory AAV and stronger co-localization with cholinergic cells is seen with the high falling rat (A and C). A schematic of the inhibitory AAV infusion target in the BF at −1.0 AP is shown in (E). Boundaries of the cell counting areas for the 3 BF subregions (nBM, SI, HDB) is also shown. For the 16 *Dual Loss ACh/DA* group rats included in the histologic analysis, viral transfection efficacy and the degree of double labeling was significantly correlated with counts in the SI as well as the HDB and nBM. In addition, the efficacy of the optogenetic virus activation, in terms of fall propensity (LED-induced falls minus no LED falls), was significantly correlated with viral transfection efficacy in the SI and HDB, but not for counts obtained from the nBM. Co-labeled cells correlated with falls in the SI (F) but not the other two structures. (G) shows a schematic of the dorsomedial striatum 6-OHDA infusion sites for the 16 *Dual Loss ACh/DA* group rats at AP +0.2. The red shaded areas depict the TH depletion spaces directly in the dorsomedial stratum (9 rats) with placement scores of 5 (9 rats) and placement scores of 4 (7 rats) with depletion spaces slightly lateral. The combined TH rating scores (average of placement and size; size scores varied between 1 and 4; AP +1.2 and AP +0.2) correlated negatively with net falls (LED – no LED) from the zig-zag rotating rod with continuous LED inhibition (H).

Optogenetic AAV infusions targeting cortically projecting neurons of the basal forebrain were conducting using the same parameters as prior AAV infusion experiments using DREADDs (Kucinski et al 2019, Kucinski et al 2022). The inhibitory AAV spread diffusely into the basal forebrain: anteriorly to cholinergic and non-cholinergic neurons of the globus pallidus and into the ventral pallidum and bed nucleus of the stria terminalis; and posteriorly through the ventromedial globus pallidus and magnocellular cholinergic neurons of the nucleus basalis of Meynert (nbM) (Zaborszky et al., 2008), as well as the cholinergic neurons of the substantia innominata (SI) and the horizontal limb of the diagonal band (HDB). The LED fibers were placed in basal forebrain at approximately AP −0.8, halfway between two infusion sites of the AAV (AP −0.6 and −1.0) (See Figure 3E for a schematic of the BF target area).

Figure 3A–D shows microphotographs from the SI of two *Dual Loss ACh/DA* Disruption group rats, one ‘high faller’ (15 falls in 24 trials from the zig-zag rod trials with continuous LED inhibition) and one ‘low faller’ (6 falls in 24 trials), exemplifying the widespread expression of the inhibitory AAV (Alexa 488; GFP, green). The magnified area shows cholinergic cells (Alexa 594; VAChAT, red) co-expressing the inhibitory AAV. The high faller had more robust expression of the inhibitory optogenetic AAV and stronger co-localization with cholinergic cells. Transfection efficacy of the AVV was estimated using manual counts of GFP-positive cells and co-labeled VAChAT-positive cells in three major regions of the BF projecting to cortical regions (nbM, SI, HDB) using the *Dual Loss ACh/DA* group (N=16) rats.

#### Neostriatal 6-OHDA lesions.

Dopamine terminals within the dorsomedial striatal projection region of the medial prefrontal (prelimbic) cortex were targeted with 6-OHDA infusions using the same parameters of previous experiments (Avila et al., 2020; Kucinski et al., 2013; Kucinski et al., 2017). TH lesion scores were graded to characterize the size and placement of the TH depletion spaces (see Figure 3 for a schematic of target spaces). TH lesions sized >2.00 mm (dorsoventral) × 0.5 mm (mediolateral) × 0.5 mm (anteroposterior) were rated highest (5) and lesions smaller than 0.1 mm x 0.1 mm received the lowest score (1). Lesions centered in and largely limited to the dorsomedial region received the highest score (5), whereas lesions lateral to this prefrontal projection region received lower grades (see Methods for further details and Figure 3G). Placement scores of the lesions were consistently high, with an average of 4.64±0.11 (out of 5). Size scores were 2.55±0.19 (out of 5), and the average overall TH score was 3.59±0.10. The TH scores of 16 *Dual Loss ACh/DA* rats significantly correlated with LED-induced falls from the zig-zag rotating rod with continuous LED inhibition (r = 0.52, p = 0.04), as in, rats with striatal TH depletion areas that tended to be more localized to the dorsomedial striatum and larger were more likely to fall with continuous LED inhibition (Figure 3H). TH scores did not correlate significantly with LED-induced falls from the straight rod with either 1s or continuous LED inhibition, nor from the zig-zag rod with 1s LED inhibition (r<0.26, p>0.33 for all).

### Relation of Histological Virus Analysis to Falls

Basal forebrain GFP cell counts were positively correlated with net falls overall on the two rods (falls in LED trials minus falls in non-LED trials over the 3-day block), but primarily revealed in performance on the zig-zag rod. In trials with the straight rotating rod with both 1s LED inhibition and continuous LED inhibition there no were significant correlations between GFP-positive or co-labeled GFP+VAChAT cell counts and net falls (GFP: 1s LED inhibition: r<0.50, p>0.06 for all subregions; continuous LED inhibition: r<0.30, p>0.25 for all). However, on the zig-zag rotating rod runs, LED inhibition with 1s pulses induced falls that correlated with GFP-positive cell counts in the SI only (r = 0.56, p = 0.02; r<0.36, p>0.17 for the HDB and nbM), but not with the co-labeled cell counts in any subregion (r<0.38, p>0.15 for all). With continuous LED inhibition on zig-zag rod trials, GFP-positive cells correlated with net falls in the SI (r = 0.58, p = 0.02) and the HDB (r = 0.64, p = 0.01) (nbM: r = 0.33, p = 0.21) and co-labeled cells correlated with net falls in the HDB (r = 0.53, p = 0.04; SI and nbM: r<0.47, p>0.07) (Figure 3F).

## Discussion

Cholinergic deficits in the cortex and BF are commonly observed in PD, particularly in individuals with high fall propensity, as well as cognitive dysfunction (Grothe et al., 2021; Schulz et al., 2018). Previous studies using a rat model of dual DA and ACh disruption indicated that striatal 6-OHDA lesions of dorsomedial dopamine terminals combined with lesion-induced losses of cholinergic BF neurons, or nonspecific DREADD inhibition BF neurons induced heightened falls in rats (Kucinski et al., 2013; Kucinski et al., 2017; Kucinski et al., 2019). Here the dual DA and ACh disruption model was extended by combining selective optogenetic inhibition specifically of ACh neurons in the BF with striatal 6-OHDA lesions to assess the effects of selective, phasic inhibitions of BF ACh projections when added to dorsomedial striatal dopamine depletion. The more challenging MCMCT balance beam task, using a rotating zigzag rod, was added to better reveal balance and coordination deficits unmasked by the dual disruption model. These results confirm that selective optogenetic inhibition of cholinergic BF neurons, when combined with striatal dopamine depletion, induces a higher propensity to fall than either disruption alone, and this propensity is revealed especially on the more challenging zig-zag rod task.

Unlike rats with ACh and DA system disruption, rats with only ACh disruption and sham striatal infusions did not exhibit increased falls with BF cholinergic optogenetic inhibition, consistent with previous findings using lesions (Kucinski et al., 2013), validating the critical importance of dual striatal DA and cortical ACh systems in modulating complex movements. It was suggested that PD cognitive and related symptoms are a dual syndrome of DA mediated fronto-striatal disruption and a dementia syndrome in part mediated by cholinergic systems (Kehagia et al., 2013). The unmasking of underlying DA deficits in patients that also develop cholinergic losses may be a primary cause of falls and the reason why L-DOPA therapy that enhances dopaminergic output does not improve falls. Pro cholinergic treatments in rat models (Kucinski et al., 2015; Kucinski et al., 2017) and human subjects (Albin et al., 2021; Quik et al., 2019; Stuart et al., 2020) suggest that such drugs may be a beneficial adjunct therapy against falls and related gait, balance, and postural impairments in PD patients with cognitive/cholinergic deficits.

### Falls by sign-trackers as well as goal-trackers.

Our current results also indicate that this dual disruption-induced propensity to fall applies equally to females and males, and also equally to sign-trackers (STs) as well as to goal-trackers (GTs). This contrasts to a previous finding that found BF disruption elevated falls primarily only in GTs, and not in STs (Kucinski et al., 2019). GTs were previously found also to have higher levels of cholinergic neuromodulation in prefrontal cortex (Koshy Cherian et al., 2017) and to perform superiorly on a sustained attention task relative to STs (Kucinski et al., 2022) compared to sign-trackers.

However, the previous study that found greater vulnerability to falls in GTs, induced by nonspecific chemogenetic BF inhibition, did not include striatal DA lesions. 6-OHDA lesions of dorsomedial striatum would interact with prefrontal systems that update habitual and goal-directed behaviors (Smith & Graybiel, 2013), and impact converging pathways with dorsolateral striatal regions involved in habits and which are affected in PD patients (Redgrave et al., 2010), and which may be relevant to ST performance when combined with BF ACh losses. Conversely, our current study explicitly assessed the effects of dual ACh/DA disruption, as well as separate ACh inhibition and 6-OHDA lesions, on vulnerability to falls.

A question may remain as to why GTs were not impaired in our study by BF ACh inhibition alone? An approach was used that differed from previous studies, which may potentially be related to why it was found that dual disruption, but no single disruption, caused elevated falls in both GTs and STs. For example, this study specifically inhibited only ACh neurons in the BF, in contrast to the nonspecific DREADD inhibition of BF neurons used in Kucinski et al., 2019. Nonspecific targeting of the BF with DREADDs may affect other neurotransmitter systems such as GABAeric neurons which could confound the role of the cholinergic system (Gritti et al., 2006). Also, CNO injections that work on the time scale of several hours may provide a more tonic inhibition with different dynamics than optogenetic inhibition. The sudden suppression of ACh neuronal activity caused by optogenetic inhibition could conceivably be more disruptive to neuronal computations needed by GTs than the relatively slow and gradual disruption induced by chemogenetic inhibition. It remains possible that GTs are more vulnerable than STs to falls induced by dual disruption that involves more gradual or milder impairment of ACh function than here. But these results suggest that both GT and STs are vulnerable to falls induced by dual ACh/DA disruption. The human population embodies a spectrum of GT and ST behaviors rather than strict categorical divisions into groups, and the higher fall propensity of both groups of rats may more realistically reflect how PD patients are affected by cholinergic system disruption.

### Types of falls.

Rats with dual DA and ACh disruption fell more with both 1s LED inhibitory pulses (given twice per 3-meter traversal) and continuous LED inhibition for the duration of traversals. A brief analysis of fall behavior did not reveal differences in types of falls or differences in falls induced by 1s LED inhibition vs continuous LED inhibition. In general, falls with optogenetic disruption appeared to be similar in nature as those observed with chemogenetic disruption (Kucinski et al., 2019). Rats fell both ‘spontaneously’ with no obvious trigger or slowed with low motor vigor and were unable to effectively rebalance and continue forward traversal following slips or missteps, leading to falls.

As in previous MCMCT experiments, females were generally faster to traverse both the straight and zig-zag rotating rods than males, and fell less frequently from the zig-zag rods across all conditions (Kucinski et al., 2017; Kucinski et al., 2019). This was likely due to males’ larger body size which makes it difficult to navigate tight bends of the zig-zagged sections and re-balance following slips or missteps. Both 1s and continuous LED inhibition increased falls over baseline in males and females on the zig-zag rotating rod in *Dual Loss ACh/DA* rats, indicating that disruption of cholinergic BF neurons impairs complex movement over a range of baseline motor abilities and body sizes.

Histological analysis verified that rats with *Dual Loss ACh/DA* disruption that were more affected by LED inhibition, particularly continuous inhibition for the duration of traversals, had higher expression of the inhibitory Optogenetic AAV in the SI and HDB of the BF. This result is consistent with expression of DREADDs that evoked falls with CNO administration (Kucinski et al., 2019). Separately, rats with larger and more specific 6-OHDA lesions of the dorsomedial striatum in the *Dual Loss ACh/DA* Disruption group tended to fall more with continuous LED inhibition, a result that is consistent with previous experiments using the dual system lesions (Kucinski et al., 2013, Kucinski et al., 2015), and similar to human patients (Bohnen et al., 2009). More severe disruptions of either the cholinergic BF or dorsal striatal dopamine system may contribute to increased movement deficits rather than these impairments being a function of disruption of a single system.

Taken together, these results support and extend previous findings in rats with striatal dopamine losses that disruption of the BF-cortical cholinergic complex precipitates falls and related gait, balance, and postural deficits, similar to human PD patients. These effects were observed consistently in both GT and ST rats with varied cognitive-motivational modes of complex surface traversal. Also, since the optogenetic inhibition reveals the impact of BF cholinergic disruption on falls on a sub-second time scale, therapies that enhance cholinergic output in such a phasic manner may potentially alleviate these dual-system movements deficits, including symptoms of cognitive decline.

## Figures and Tables

**Figure 1.**
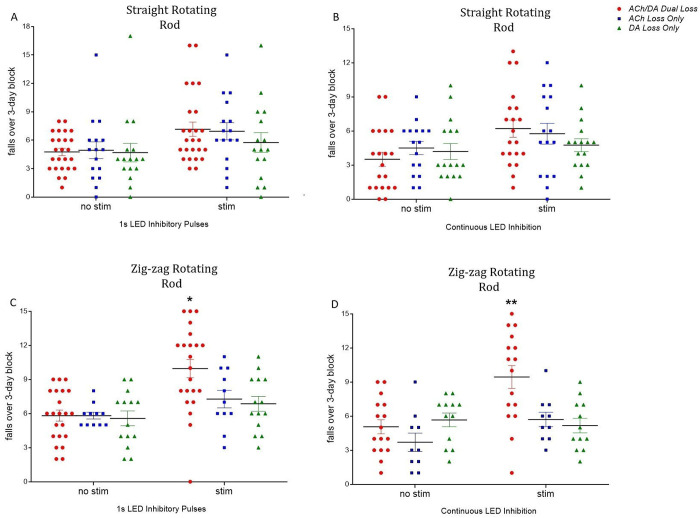
Fall totals on each rotating rod 3-day test block. Since falls did not significantly differ between days within any condition, the combined 3-day total out of a possible 24 falls in each 3-day test block is shown per rat. (A) Straight rotating rod, 1s LED inhibitory pulses (N=57). There were elevated falls overall in LED runs but no group differences. (B) Straight rotating rod, continuous pulses (N=52). Falls were again elevated with continuous LED inhibition, but the source of a significant LED x group interaction was not located. (C) Zig-zag rotating rod, 1s LED inhibitory pulses (N=47) and (D) Zig-zag rotating rod, continuous LED inhibition (N=37). Rats with *Dual Loss ACh/DA* fell more than *ACh Loss Only* or *DA Loss Only* groups with both 1s and continuous LED inhibition. *P<0.05, **P<0.01, more falls in *Dual Loss ACh/DA* group than the *ACh Loss Only* or *DA Loss Only* groups, *posthoc* LSD, after significant group x LED interaction.

**Figure 2.**
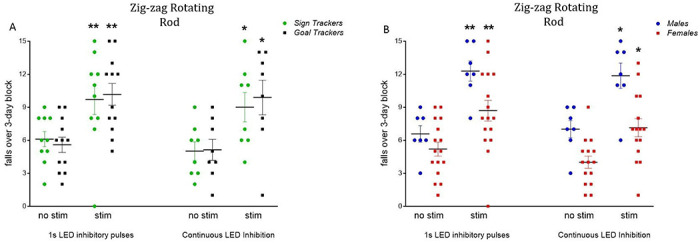
(A) Falls by GTs vs STs in the *Dual Loss ACh/DA* group from the zig-zag rotating rod, with 1s LED inhibitory pulses and continuous LED inhibition. GTs (N=10) and STs (N=12) were both impaired by LED inhibition of BF cholinergic neurons. (B) Male rats fell more than females on both zig-zag rotating rod conditions; however, this did not interact with Disruption group or LED stim in either condition. The number of falls by female (N=15) and male (N=7) from the zig-zag rotating rods with 1s and continuous LED inhibition are shown for *Dual Loss ACh/DA* rats. *P<0.05, **P<0.01 pairwise comparisons.

**Table 1.** Number of Chat-Cre positive rats undergoing PCA screening and Goal Trackers (GTs) and Sign Trackers (STs) used to assess MCMCT falls from the straight and zig-zag rods with continuous LED inhibition (f, females).

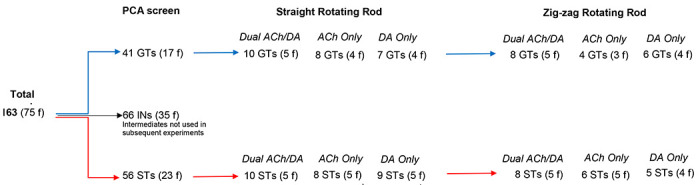

**Table 2.** MCMCT Training and Testing Sequence

Day	Traversal condition	Number of traversals
1	Plank	Training: 6 traversals
2	Straight rod stationary (0 rpm)	Training: 6 traversals
3	Straight rod rotating (3-8 rpm)	Training: 6 traversals
4	Straight rod rotating (8 rpm)	8 traversals (4 no stim, 4 with 2 inhibitory LED pulses)
5	Straight rod rotating (8 rpm)	8 traversals (4 no stim, 4 with 2 inhibitory LED pulses)
6	Straight rod rotating (8 rpm)	8 traversals (4 no stim, 4 with 2 inhibitory LED pulses)
7	Zig-zag rod stationary (0 rpm)	Training: 6 traversals
8	Zig-zag rod rotating (3-5 rpm)	Training: 6 traversals
9	Zig-zag rod rotating (5 rpm)	8 traversals (4 no stim, 4 with 2 inhibitory LED pulses)
10	Zig-zag rod rotating (5 rpm)	8 traversals (4 no stim, 4 with 2 inhibitory LED pulses)
11	Zig-zag rod rotating (5 rpm)	8 traversals (4 no stim, 4 with 2 inhibitory LED pulses)
12	Straight rod rotating (8 rpm)	8 traversals (4 no stim, 4 with continuous LED inhibition)
13	Straight rod rotating (8 rpm)	8 traversals (4 no stim, 4 with continuous LED inhibition)
14	Straight rod rotating (8 rpm)	8 traversals (4 no stim, 4 with continuous LED inhibition)
15	Zig-zag rod rotating (5 rpm)	8 traversals (4 no stim, 4 with continuous LED inhibition)
16	Zig-zag rod rotating (5 rpm)	8 traversals (4 no stim, 4 with continuous LED inhibition)
17	Zig-zag rod rotating (5 rpm)	8 traversals (4 no stim, 4 with continuous LED inhibition)
